# Tailoring the delivery of cancer diagnosis to adolescent and young adult patients displaying strong emotions: An observational study of two cases

**DOI:** 10.3402/qhw.v11.30763

**Published:** 2016-04-27

**Authors:** Live Korsvold, Hanne Cathrine Lie, Anneli Viktoria Mellblom, Ellen Ruud, Jon Håvard Loge, Arnstein Finset

**Affiliations:** 1 Department of Behavioural Sciences in Medicine, Institute of Basic Medical Sciences, Faculty of Medicine, University of Oslo, Oslo, Norway; 2 Department of Paediatric Medicine, Women and Children's Division, Oslo University Hospital, Oslo, Norway; 3 National Resource Center for Late Effects after Cancer Treatment, Oslo University Hospital, Radiumhospitalet, Oslo, Norway; 4 Regional Centre of Excellence in Palliative Care, Oslo University Hospital, Oslo, Norway

**Keywords:** Delivering bad news, oncology, AYA, clinical communication, case study

## Abstract

Delivering the bad news of a cancer diagnosis to adolescent and young adult (AYA) patients who display strong emotions is particularly challenging not the least because AYAs are at a vulnerable developmental stage. Due to the lack of research on how to personalize the delivery of bad news to AYA patients’ emotions we report a case study of the communicative behavior of oncologists in two such consultations to describe the complexity of the phenomena at study. We audio-recorded and transcribed consultations where oncologists delivered cancer diagnoses to nine AYAs aged 12–25 years. Two of these patients displayed particularly strong emotional behavior (anger, fear, and sadness) and were chosen as cases. An interpretative analysis in three steps was applied to investigate the oncologists’ communicative behavior when delivering bad news. The focus was on how the oncologists responded to the strong but different emotional behaviors of the AYAs. We also related the oncologists’ communicative behavior to elements from a widely used protocol for delivering bad news. We found that the oncologists applied five communication strategies: elicit patient perspective, provide information, respond to patient's expression of emotion (acknowledging and containing emotions), encourage commitment to treatment, and provide hope. The findings illustrate how oncologists’ communicative behavior may be tailored to individual expressions of emotions in AYA cancer patients.

Giving diagnostic information on oncology is a challenging task. It has been characterized as being “on the edge of rationality” for both patient and doctor as the bad news may provoke strong emotional reactions in the patient (Maynard & Frankel, 2006, p. 277). There is evidence that the way in which adverse diagnostic information is delivered may influence patients’ anxiety and stress (Schofield et al., 2003) and may also impact their satisfaction with care, adherence to treatment, and other health outcomes (Fallowfield & Jenkins, 2004).

A diagnosis of cancer is devastating for anyone but perhaps particularly for adolescents and young adults (AYAs), as they are in a particularly vulnerable developmental stage (Keegan et al., 2012; Kent et al., 2012; Zebrack et al., 2014). At a time in life when they would expect to be making the transition to independence they become dependent on others because of a life-threatening disease (Kent et al., 2012). Delivering a cancer diagnosis to AYAs is therefore a delicate and challenging task for oncologists.

By bad news we mean “any information that produces a negative alteration to a person's expectations about their present and future” (Fallowfield & Jenkins, 2004, p. 312). A cancer diagnosis is an obvious example of bad news. In oncology, two factors complicate the delivery of the diagnosis. First, there may be limited time available to explain the implications of the diagnosis and the treatment options because the medical condition of the patient may require immediate treatment. Second, the giver and the receiver of the diagnosis often meet without substantial previous contact (Fallowfield & Jenkins, 2004).

Most research into delivery of bad news has been descriptive, in the sense that communication patterns during the giving of diagnostic information are described and discussed (Maynard & Frankel, 2006), or based around the development of guidelines and strategies for delivering bad news (Baile et al., 2000; Girgis & Sanson-Fisher, 1995). The most widely quoted guideline in oncology, a six-step protocol for delivering bad news: SETTING up the interview (S); assessing the patient's PERCEPTION of his or her condition (P); obtaining an INVITATION to (I); providing KNOWLEDGE and information to the patient (K); responding empathically to the patient's EMOTIONS (E), and explaining the treatment STRATEGY and providing a SUMMARY of the interview (S), is SPIKES (Baile et al., 2000). SPIKES is based on expert opinion and published by the American Oncologists Association (Baile et al., 2000).

The existing guidelines, including SPIKES have been criticized for not acknowledging the need for a flexible approach and the variety of situations in which bad news is delivered (Eggly et al., 2006). In a meta-synthesis of qualitative studies of breaking bad news in oncology Bousquet et al. (2015) highlighted that one of the communicational challenges in the patient–oncologist encounter was to deal with emotions. Studies have also shown that it is important to deliver bad news in an empathic manner because this reduces the patient's distress and promotes recall of the information given (Van Osch, Sep, Van Vliet, Van Dulmen, & Bensing, 2014). Moreover, patient's value that health care providers acknowledge their reactions and recognize the gravity of the bad news (Back et al., 2011; Fujimori & Uchitomi, 2009; Shaw, Dunn, & Heinrich, 2012). There are individual differences in patients’ preferences regarding handling of emotions (Bousquet et al., 2015). It appears important to tailor the providers’ communication to the individual patient. Still, little is known of how oncologists preform this tailoring in real time consultations. Maynard and Frankel (2006) studied the delivery of good news and bad news in real time consultations, but they did not focus on the patients’ expressions of emotions and how this might influence the delivery of medical news.

Most studies on delivering bad news in cancer care and other medical settings have involved adult patients (Baile et al., 2000) or children (Farrell, Ryan, & Langrick, 2001). We identified only one study with a specific emphasis on adolescents’ perspectives on receiving a cancer diagnosis (Stegenga & Ward Smith, 2009) and the focus of this study was the lived experience of being diagnosed with cancer, rather than the communication of the diagnosis. Delivering bad news to AYA patients may pose a particular challenge. Miedema, Hamilton, and Easley (2007) found that AYA survivors had not felt prepared to face a life-threatening disease at their stage of life. Adolescence is a time of strong, often negative, emotions (Arnett, 2014). Results from neuroscience indicate that the traditional subdivision in physical, psychological, and social development might disguise some important insights of the interrelatedness of these developmental domains during adolescence and young adulthood. The results point to how the physiological development and hormonal changes of the brain during adolescence and young adulthood can explain much of the psychological and social development going on at the same time (Casey, Jones, & Hare, 2008). Casey et al. (2008) described how adolescents have a “heightened responsiveness to incentives and socioemotional contexts during this time, when impulse control is relatively immature” (p. 111). Both the physiological development and the hormonal changes contribute to decreased goal-oriented behaviors, organization, planning and impulse control in AYAs compared to adults (Casey et al., 2008; Giedd, 2008; Steinberg, 2010). This may affect AYAs communication style (Colver & Longwell, 2013). The oncologist giving a diagnosis to an AYA may be faced with a strong emotional behavior indicating concern (sadness, surprise, fear, anger, disgust, or shame) (Ekman et al., 1987) and should be prepared to tailor his or her communication style to the individual AYA patient (Stegenga & Ward Smith, 2009). There is, however, a lack of research on how to tailor the delivery of bad news, such as a cancer diagnosis, to AYA patients in general and to AYAs displaying strong emotions in particular. Knowledge about this can be useful for clinicians to be able to personalize communication in this challenging clinical setting (Bousquet et al., 2015).

Our analysis focuses on the pragmatic aspects of communication, that is, the behavioral and relational aspects of communication as opposed to syntax and semantics per se (Watzlawick, Bavelas, & Jackson, 1967). The aim of the present paper thus is to investigate how oncologists delivering cancer diagnoses to AYAs tailored their communication to the patients’ expressions of strong emotions (anger, fear, and sadness). We investigated to what extent and how oncologists not only presented medical information in a one-way mode but also engaged in personalized communication behavior with explicit references to the individual patient and possibly also to himself or herself and to the doctor–patient relationship when delivering a cancer diagnosis to AYA patients displaying strong emotions.

## Method

### Design

The design of the current paper is interpretive and it is based on a pragmatic view of research implying that the research question leads the method and that multiple methods of data collection and analysis can be appropriate to answer this question (Creswell, 2013a, 2013b). We could not find any research on how physicians tailor communication to AYAs displaying strong emotions, therefore we decided to do an in-depth study of communication in real-time consultations. Strong emotions can have different displays, and we applied instrumentive and collective case-study design to be able to investigate the complexity of the issue at study (Creswell, 2013a): how oncologists delivering cancer diagnoses to AYAs tailored their communication to the individual patients’ expressions of strong emotions (anger, fear, and sadness)? Yin (2014) supports that such relationship issues are relevant for case studies. Flyvbjerg (2006) holds “that a discipline without exemplars is an ineffective one” (p. 242). We aim to contribute to the discipline of communication in AYA medicine by conducting a case study that provides exemplars of the complex practice of delivering cancer diagnoses to AYA cancer patients displaying strong emotions.

### Participants

The two cases were taken from a sample of nine audio-recorded consultations from three different hospital wards in Oslo, Norway, in 2012–2013. The nine consultations were sampled to study communication in medical consultations in which oncologists informed AYA patients (12–25 years of age) about a cancer diagnosis and the proposed treatment.

Patients of both sexes were eligible if they were between 12 and 25 years of age at the time of inclusion and were about to be informed about a cancer diagnosis and a subsequent treatment proposal. Moreover, the included patients were required to have adequate Norwegian language proficiencies (no interpreter needed) and to be able to provide informed consent.

We recruited patients from one pediatric ward (aged 12–18 years) and two medical wards for adult patients (aged 19–25 years) at Oslo University Hospital. Eleven patients were invited to participate. Of these patients, one patient refused to participate and one patient terminated the audio recordings and withdrew her consent. A number of eligible patients were not included in the study because of recruitment or logistical problems due to the short timeframe between the patients’ arrival at the hospital and the consultations. The exact number of “lost” patients is unknown because the patients often had their treatment at other wards. Patients were recruited to the study between September 2012 and January 2014. The recruitment of patients was challenging, but the audio recordings include the desired age span, female and male patients, and the most common diagnoses at the three different hospital wards. Data collection ended when we considered the amount and quality of the data to be sufficient.

Both audio-recorded consultations took place in the patients’ rooms at the oncology wards. In both consultations between oncologist and patient, the patient's mother and a nurse were also present.

The participating oncologists administered the audio recording of the consultations using a small digital voice-recorder placed on the table. We felt the patients had a right to get a copy of their own consultation therefor they received an electronic copy (mp3 format) of his or her consultation about a week later.

The consultations were transcribed verbatim. Profanities were transcribed as “f…” or “s…”; hesitations were transcribed with “eh” or “ehm.” Audible nonverbal communication such as pauses and crying has been noted in parentheses. Pauses lasting 2 s or longer are specified in the text. The lines of dialog presented in Tables II–VII are numbered.

### Sampling of cases

Although the issue in the current paper pertained *to the tailoring of the news*, we sampled on AYAs displaying strong emotions. For the current paper we chose two cases following the principles of purposeful intensity sampling (Patton, 2002). Intensity sampling requires some information about potential participants and considerable judgment because it consists of selecting a sample that provides an intense manifestation of the phenomenon of interest (Patton, 2002). LK and AF, using their experience in clinical pediatric oncology nursing (LK), clinical psychology (AF), and communication research (AF), chose the two consultations in which the patients—in one case a male patient with sarcoma and in the other a female patient with leukemia—exhibited a strong, explicit emotional behavior, that is, anger, fear, or sadness. In case study research, one should select data that enable a study of the complexity of the research question and also triangulation of the observations (Hyett, Kenny, & Virginia Dickson-Swift, 2014). Therefore, cases in which the patients displayed contrasting patterns of highly challenging, intensely emotional behavior were chosen.

### Background information of the two cases

In both cases, the patients had been told in previous consultations that they might have cancer; however, it was in the consultations that we analyzed that they were given a cancer diagnosis and the proposed treatment was explained. In both cases there was still uncertainty about the exact diagnosis, but the oncologist wanted to start treatment because they had identified the main type of disease, sarcoma (case A), and leukemia (case B). Both oncologists were in their late thirties and worked in adult oncology.

### Analysis

The analysis was an interpretive process in three steps inspired by (Creswell, 2013a). One feature of the analysis is that *Discussion* is part of the analyzing process (the third step).

First, LK and AF listened to the consultations several times and read the transcripts, independently, in order to identify sequences related to displays of emotional behavior by the patients and how the oncologists responded to these emotional displays. With the information about the aim of the study, the patients and context in mind and in order to triangulate the interpretations, the two analysts (LK an AF) separately identified sequences where the oncologists’ communication seemed to be of significance in handling the emotional expressions. For example, both analysts identified sequences of strong expressions of emotion (anger, fear, and sadness) that in some way seemed to affect how the oncologists “did” empathy in the two consultations. Second, the identified sequences were compared and studied in more detail, that is, the example of the different ways to “do” empathy was one of the elements of the oncologists’ communicative behavior that seemed relevant to grasp the complexity of the phenomenon at study, see *Results*. To further reveal the complexity of the cases, the case study report should contain a description of the context of the case (Creswell, 2013a; Hyett et al., 2014), of how it occurs in real-time, and of “the themes or issues that the researcher has uncovered in studying the case” (Creswell, 2013a, p. 99). In *Results*, the collective context of the two cases as well as the individual contexts of each case is described. Furthermore, to give a sense of story to the presentation (Hyett et al., 2014), the chosen sequences and the analysis of them is presented chronologically for each case. Finally, the third step of the analysis is presented in *Discussion* where we “analyzed for similarities and differences” (Creswell, 2013a, p. 99) across the two cases. Here, three elements of the oncologists’ communicative behavior in the two cases (three overall themes) are discussed. Because the SPIKES protocol (Baile et al., 2000) is so widely used but also because it is criticized for simplifying the delivery of bad news, we chose to relate our findings with the elements in the protocol in *Results*. This was not done as an analytical tool in *Results* but to enable further inquiry of how the oncologist communicative behavior relate to the elements in the SPIKES protocol in *Discussion*. In *Discussion*, we did an analysis of the overall meaning of the cases and ended up with a conclusion about what people can learn from the case study also called “naturalistic generalizations” (Creswell, 2013a, p. 200).

To ensure the reliability of the analysis we also triangulated the analyses of the two cases by involving all the authors (AVM, HCL, EL, and JHL) in discussions of the interpretations and decisions of presentation. To further explore different ways to interpret the cases, we presented and discussed the analysis of the cases in academic forums.

### Ethics

The Regional Committee for Medical and Health Research Ethics approved the study (reference number 2011/2290). One of the nurses at the wards or the first author (LK) invited the patients, family members, physicians, and nurses to take part in a study in order to investigate real-time communication between AYA cancer patients and health-care providers, and asked for signed consent to audio record the scheduled consultation. The participating patients, parents, oncologists, and nurses provided written consent. The participants had the right to withdraw at any time. To ensure participant confidentiality in the recorded consultations only the members of the research group have listened to the tapes. Personal information about the participants that was not necessary to the transparency of the analysis has been left out to ensure confidentiality in this paper.

## Results

### Case A: high school male student with sarcoma expressing frustration and anger

At the time of the recorded consultation, the patient had been hospitalized for 3 days. He had previously been admitted to two other hospitals and had undergone multiple tests and examinations to investigate his painful symptoms. At the time of the consultation, the patient was in great pain, and during the consultation, the oncologist informed him that the medical team no longer only presumed but had firmly established that he had a large sarcoma. Eight minutes into the consultation the patient started to express doubts about whether the medical team could help him. The recorded consultation lasted for 21 min.

Early in the consultation, the oncologist explained the results of the CT examination and responded to questions from the patient's mother. The patient responded with sarcastic remarks indicating dissatisfaction with how long the assessment phase had taken. The oncologist concluded by informing the patient that no further examinations would be needed and that they had to wait for all the test results to arrive. So far, the findings indicate a tumor that will respond to chemotherapy (Table I).

**Table I:** Case A, line 1–10.

**1**	Patient:	Why not just remove the f(….) shit?
**2** **3** **4** **5** **6**	Doctor:	Yes, that's a good question. If we remove it, as it is, hem, operate it out, it is important that we can do the surgery in a way to make sure that we include all, and in the pelvis, it is cramped. It is difficult to get to; there is much else that is nearby. So that if we can shrink the tumor with chemotherapy, then surgery will be much easier and the results will be better.
**7** **8**	Patient:	So just sit and wait until it has grown big and you do not have a chance in the world to operate it out
**9** **10**	Doctor:	If we are not starting up with chemo now, then you need surgery fast. It is not an option to wait.

In this excerpt, the patient indirectly expressed anger and despair by way of profanities (line 1) and sarcasm (lines 7–8) and also expressed a rather negative attitude to the oncologist. The oncologist, however, did not appear to be influenced by the patient's rudeness. In lines 2–6 and again in lines 9–10, he calmly provided information on why it was considered best to start chemotherapy before surgery (“K” for knowledge in the SPIKES protocol) (Baile et al., 2000).

The patient went on to indicate a wish to seek treatment abroad to speed up surgery, but the mother expressed trust in the medical team. In line 11, Table II, the patient expressed his worry that the tumor will carry on growing:

**Table II:** Case A, line 11–22.

**11**	Patient:	But it grows.
**12** **13** **14**	Doctor:	I realize that you think this takes too long, John. I can only promise you that we do things as quickly as possible, but, but, but to you this takes too long. We understand.
**15**		Pause 5 s
**16**	Mother	Mm
**17**	Doctor:	If we start chemotherapy tomorrow are you in to it?
**18**	Patient:	How long does it take before I am f(…) bald on the head then?
**19**	Doctor:	Excuse me, what did you say?
**20**	Patient:	How long does it take before I'm f(…) bald on the head?
**21**	Doctor:	It takes a few weeks.
**22**		Pause 12 s

In line 12, we see a clear shift in the oncologist's communication away from mainly using a rather formal “we” mode and responding to the content of the patient's utterances; here he said, “I realize that you think this is taking too long, John,” demonstrating a more personal engagement and showing empathy (“E” for empathy in SPIKES) (Baile et al., 2000), acknowledging that the patient had reason to be impatient. Although he did not explore the patient's emotion he remained patient, using pauses in lines 15 and 22 to give the patient space to react. In line 17, the oncologist explicitly asked if the patient was up for starting treatment (“S” for summary in SPIKES) (Baile et al., 2000). The patient did not answer the question about willingness, instead he asked about hair loss in line 18.

At this point, the oncologist went on to say that they needed to give the patient a Central Venous Device (CVD) to give the chemotherapy safely. He said that they would insert it the next day. The patient's reaction is given in Table III.

**Table III:** Case A, line 23–31.

**23** **24**	Patient:	We are talking about being tortured before you must die. I am f(…) aware that I will die, but damn I need to be tortured first.
**25**	Doctor:	Our plan is *not* that you will die.
**26**	Patient:	But that is what is happening.
**27** **28** **29** **30**	Doctor:	So far, what we know is that the disease you have is a tumor in the abdomen and that we can, can treat, ehm. So it's our goal that we will treat it and you will get well, eh but we need you to be up for it. If not, it is no use in doing what we plan to do.
**31**	Patient:	No, I'm in but

In lines 23–24, the patient once again expressed his skepticism that the medical team would be able to help him and said explicitly “I am f(…) aware that I will die.” The oncologist continued his straightforward responses and addressed the content and not the emotional component of the patient's utterances (line 25). In lines 27–30, the oncologist once again raised the issue of the patient's readiness to proceed, saying: “We need you to be up for it. If not there is no use in doing what we plan to do.” The patient gave his consent in line 31: “No, I'm in but.”

After a summary of the conclusions and decisions from the consultation and a few questions and responses, the consultation ended.

### Case B: young woman with leukemia expressing sadness and despair

Two days prior to the recorded consultation, the patient had a scheduled pregnancy follow-up appointment with her GP. She was pale and tired, but she thought it was due to her pregnancy. The GP ordered blood tests. Because the hematological findings (white cell count, hemoglobin and platelet count) were abnormal, the patient was referred to the hospital for further tests. Prior to the recorded consultation, the patient had been told she probably had leukemia. She had also been told that it would be necessary to terminate the pregnancy because of the negative effects of chemotherapy on the fetus. The recorded consultation lasted for 9.5 min.

The oncologist started the consultation by eliciting the patient's perspective, asking if the patient had “thought any more about what happens next?” (An illustration of “P” in the SPIKES protocol (Baile et al., 2000)) (Table IV):

**Table IV:** Case B, line 32–37.

**32**	Doctor:	Yes, ehm, have you thought any more about what happens next? Mm
**33**		Pause 2 s
**34**	Patient:	I will terminate the pregnancy (with a weak and suffering voice)
**35**	Doctor:	Yes
**36**	Patient:	Today?
**37**	Doctor:	Yes, it will be today.

The oncologist went on to discuss the abortion procedure but returned quickly to the patient's perspective (Table V):

**Table V:** Case B, line 38–45.

**38**	Doctor:	So, that is maybe what you are—what you think about the most?
**39**	Patient:	Mm
**40**	Doctor:	Just now?
**41**	Patient:	Then I think about those eggs. Are they to be frozen down?
**42** **43** **44**	Doctor:	No, do you know what, I discussed it with the other doctors and with the cancer that you have, the cancer cells are all around in your body because the blood is all around in your body.
**45**	Patient:	That is what I thought.

The oncologist continued by giving information about the deep-freezing of ovarian tissue and concluded, “There are some patients who stay fertile anyway, but it is difficult to say in advance.” The patient's reaction is as follows in Table VI.

**Table VI:** Case B, 46–75.

**46**	Patient:	But I am not that lucky, you know.
**47** **48**	Doctor:	(Exhales through her nose) do you think like that because you have had so much trouble getting pregnant?
**49**		(The patient does not answer but it sounds like she is nodding).
**50**	Doctor:	Yes, yes I can understand that you are devastated about that, you know.
**51**	Patient:	(Sobs)
**52**	Doctor:	Mm
**53**	Mother	(Say something in another language—sounds very sad.)
**54**	Patient:	(Cries, it sounds like the mother tries to comfort her, the patient gets a tissue paper)
**55**		Pause (23 s)
**56**	Doctor:	And this that you have wanted for so long.
**57**	Patient:	(Mumbles)
**58**	Doctor:	Mm
**59**	Patient:	(Cries)
**60**		Pause (7 s)
**61**	Doctor:	But I think there is a big chance this will work out.
**62**	Mother:	She must think about herself first.
**63**	Patient:	(Cries)
**64** **65**	Doctor:	Yes, and then we'll start up with the first round of treatment, and then we'll see if we can freeze tissue after that.
**66**	Patient:	(Sobs), ehm (from high to low tone)
**67** **68**	Doctor:	This is a disease that usually responds very well to chemotherapy (with a comforting voice)
**69**	Patient:	(Sobs)
**70** **71**	Doctor:	So there is a big chance that you will be cancer-free when we get to the time when it is possible to freeze.
**72**	Patient:	(Sobs, mumbles)
**73**		Pause (15 s)
**74** **75**	Doctor:	So, do you want to know a little bit now about what we think further tomorrow, or? Mm?

In lines 51, 54, 59, 63, 66, 69, and 72, we see how the patient expressed strong emotion: she talked in a weak, pained voice, cried, and sobbed. The oncologist responded by giving detailed information about the abortion procedure, the possible harvesting of ovarian tissue and finally about the diagnosis and treatment (“K” for knowledge in SPIKES), not included in dialog samples (Baile et al., 2000). Maybe more striking than the oncologist's approach to information giving is her empathic communication, the “E” in SPIKES (Baile et al., 2000). Her response to the patient's emotional behavior was explicitly empathetic, “yes, yes I can understand that you are devastated about that, you know” (line 50) and “and this is what you have wanted for so long” (line 56). She also explored the patient's emotions (lines 47–48) by asking, “Do you think like that because you have had so much trouble getting pregnant?.” Like the oncologist in case A, the oncologist in case B also showed patience and left long pauses to open up space for the patient's reaction (lines 55, 60, and 73). Moreover, the oncologist also expressed hope, saying “…there is a good chance this will work out” (line 61), “this is a disease that usually responds very well to chemotherapy” (line 67), and “so there is a good chance that you will be cancer-free when we get to the time when it is possible to freeze” (lines 70–71). Again like the oncologist in case A, the oncologist in case B used the personal pronoun “we” when she was giving medical information but the more personal “I” when she was expressing empathy (Table VII):

**Table VII:** Case B, line 76–88.

**76**	Doctor:	Is there anything else you wonder about?
**77**	Patient:	No
**78**	Doctor:	More questions will probably arise later.
**79**	Patient:	Mm, (sobs)
**80** **81**	Doctor:	So, just take it when it comes (pause, 3 s). It is good you have your family here, then.
**82**	Patient:	Yes, it is important with support.
**83**	Doctor:	Mm. It is very much for you in a short day.
**84** **85**	Patient:	Yes, it is (sniffles and mumbles, pause 3 s) I think so, I will get through this (at the verge of tears).
**86** **87**	Doctor:	Yes, you will do that, for sure. It is a huge shock now, but then the days go by and then you will manage very well, I'm sure about that. Okay?
**88**	Patient:	Mm, (sobs)

In the final excerpt, we see that the oncologist continued her empathic communication by saying “Mm. It is very much for you in a short day” (line 83) and “yes, you will do that, for sure. It is a huge shock now, but then the days go by and then you will manage very well, I'm sure about that. Okay?” (lines 86–87). In case B, the oncologist did not explicitly ask for the patient's consent to start treatment, but the patient implied it herself saying “yes, it is (sniffles and mumbles, pause 3 s) I think so, I will get through this (on the verge of tears)” in line 84–85 after the oncologist had given information in an empathic way.

The oncologist summarized by saying that the patient will need to have the abortion first and that when they receive the results of all the tests she would tell the patient exactly what her treatment would involve. This brought the consultation to an end.

## Discussion

This inductive qualitative analysis of communication between two AYA oncology patients displaying strong emotions and their oncologists illustrates the complexity of challenges that may arise in the treatment of AYA patients.

Both patients’ behavior was characterized by strong emotional displays. They both expressed their emotions indirectly, with few if any explicit verbal expressions of emotion. The patient in case A displayed his emotions in ways typical of adolescents, using profanities and sarcasm which are seen less often in more mature patients (Arnett, 2014). The patient in case B, who found herself in a life crisis specific of a young female patient, faced with concerns about a forthcoming abortion and future reproductive potential (Knight et al., 2015), expressed her emotions nonverbally.

Moreover, both patients had strong preferences for immediate interventions. The patient in case A wanted an immediate operation and had little tolerance for the strategy chosen by the oncologist (chemotherapy before surgery), while the patient in case B preferred her eggs cells to be conserved as soon as possible.

In this discussion, we focus on the relational aspects of communication and on the behaviors, skills, and strategies that the oncologist apply to tailor the communication to the individual patients. We have identified five such behaviors that may be described in terms of interactive tasks to be performed by the oncologist, more or less corresponding to elements in the SPIKES protocol (Baile et al., 2000). First, one of the oncologists actively elicited the patient's perspective in order to handle the strong emotions of the patient (Eliciting Patient Perspective in the SPIKES protocol). Second, both oncologists provided information to the patients. In this information, (knowledge in the SPIKES protocol) the oncologists did not refer to the patient or him/herself. Third, both oncologists tailored their empathic response to the individual patients’ emotional displays by acknowledging and containing patients’ emotions (empathy in the SPIKES protocol). Fourth, the oncologists not only provided information about treatment but also took pains throughout the consultation to ensure that the patient, his or her strong emotions notwithstanding, understood and accepted the proposed treatment (strategy and summary in the SPIKES protocol). And finally, fifth, the oncologists promoted hope tailored to the emotional displays of the individual patient (strategy and summary in the SPIKES protocol). In Tables I–VII, the interactive tasks performed by the oncologists are referred to in brackets. Interestingly, the oncologists’ communication involved frequent shifts in choice of personal pronouns, shifting from the first person plural to, as well as frequent uses of second person (you), the first person singular to communicate personal engagement and emphasis on the doctor–patient relationship in the dialog (Table VIII).

**Table VIII:** Strategies and skills in oncologists’ communication in delivering diagnosis and discussion of treatment.

Strategy or skill	Interactive task performed	Use of personal pronouns	Case A	Case B	SPIKES model
Elicit patient perspective	Doctor asks patient his or her perspective	The oncologist in case B applies YOU to elicit the patient's own understanding	*(The oncologist does not elicit patient understanding before information giving)*	Have you thought more about what happens next? (line 32)	P for perception
Provide information	Doctor does not refer to the patient or to him/herself	The pronoun WE (meaning we the medical team) to give information about condition and treatment	If we can shrink the tumor with chemotherapy (line 5–6)	And then we'll see if we can freeze tissue after that (line 64–65)	K for Knowledge
Respond to patient's expression of emotion	Doctor acknowledges the emotion of the patient with reference to him/herself	Both I (to denote the oncologist's own understanding and personal engagement) and YOU (to acknowledge the patient's thoughts and feelings) are used to express empathy	I realize that you think this takes too long, John … (line 12)	Yes, yes, I can understand that you are sad about that, you know (line 50)	E for Empathy
	Doctor patiently endures and contains the patient's emotion	No specific use of personal pronouns	*Calm and patient response to anger and profanities, long pauses, somewhat contained expressions of hope*	*Calmness and long pauses*	
Encourage commitment to treatment	Doctor asks patient to actively commit him/herself to treatment	WE is used to present treatment options, but both oncologists also use YOU to engage the patient in decision of treatment (A) or to convey hope (B)	… are you in to it? (line 17) … but we need you to be up for it (line 29)	*(The oncologist does not explicitly encourage commitment)*	S for Strategy (and summary)
Provide hope	Doctor presents an optimistic projection of the effect of treatment on the patient (not only in general terms)	Whereas the oncologist in case A applies I to convey hope, the oncologist in case B uses WE, but adds YOU to make it more personal	Our plan is NOT that you will die (line 25) So it's our goal that we treat you and that you will get well (line. 28–29)	I think there is a good chance this will work out (line 61) So there is a big chance that you will be cancer-free when we get to the time when it's possible to freeze. (line 70–71)	

Elicit the patients’ perspective: The P for patient perception in SPIKES was only seen in case B. The interactive task performed by the oncologist in case B was to elicit the patient perspective. The patient was referred to directly and in a personal way as “you”: “…have you thought more about what happens next?” (line 32).

Provide information: Skelton, Wearn, and Hobbs (2002) reported that the personal pronoun “we” was used more often than “I” in medical consultations; “we” was used in talking about actions, for instance when recommending a particular treatment, whereas “I” was followed by words referring to the oncologist's thoughts. We observed something similar in our recorded consultations (Table VIII). The oncologists used the first person plural “we” when they provided information about medical assessments and treatment plans: “If we are not starting up with chemo now, then you need surgery fast.” In this strategy, the interactive task performed by the oncologists did not refer to the patient or the oncologists as him/herself but to the medical team (we). This reference to the medical team (we) might be a way for the oncologist to reassure the patient that the oncologist is not alone doing medical deliberations. Nevertheless, we suggest that oncologists’ use of the impersonal “we” can also be understood as a way of distancing themselves from personal engagement and responsibility.

### Acknowledge patients’ emotion

Both oncologists responded explicitly to the patient's expression of emotion. They acknowledged the emotion of the patient with reference to themselves and the patient by using the more personal first “I” and second person “you” when they made empathic statements: “I realize that you think this takes too long……to you this takes too long” (case A, lines12 and 13), and “Yes, yes I can understand that you are devastated about that” (case B, line 50).

In a recent discussion of the SPIKES protocol, Van Vliet and Epstein (2014) pointed out severely ill patients need to both *know and understand* and *to feel known and understood* in clinical communication. The latter requirement implies that clinical staff must be competent in handling emotions, and Van Vliet and Epstein suggested a special emphasis on empathy in the process of delivering bad news to patients with severe disease. The NURSE model (Name the emotion; Express understanding; Respect, Support, and Explore the emotion) (Pollak et al., 2007; Smith & Hoppe, 1991) is a guideline, which places more emphasis on empathy than the SPIKES protocol (Baile et al., 2000). Both the SPIKES (Baile et al., 2000) and the NURSE (Pollak et al., 2007; Smith & Hoppe, 1991) protocols emphasize the importance of the doctor responding in an empathic manner and provide examples of how to demonstrate empathy verbally: (“I am sorry that the x-rays show…”) and behaviorally (“moves your chair closer….”). The “E” in SPIKES prompts the doctor to acknowledge major emotions as they arise (Baile et al., 2000). The NURSE model additionally encourages the clinician to respect the patient's emotions (Pollak et al., 2007; Smith & Hoppe, 1991).

In our data, we observed how emotions were acknowledged in both cases but with an explicit naming and expression of understanding of specific emotions only in case B (“I understand that you are devastated…” (line 50)). In her theory of suffering, Morse (2001) points out that a patient in a state of emotional suffering who is displaying his or her emotions openly requires comfort, and both active acknowledgment of emotions and expressions of hope may be comforting the patient.

### Contain patients’ emotions

A strategy of actively *acknowledging* emotions was supplemented by more passively *containing* the emotional expressions and leaving long pauses that opened up for the patient to react, most notably seen in case A. The patient in case A did not express his emotions explicitly, but they were implicitly expressed in his use of profanities, sarcasm, and expressions of distrust. The oncologist patiently tolerated the patient's outbursts (“Yes, that's a good question,” line 2) and gave respectful comments in a matter-of-fact way. Half way into the consultation, he markedly altered his communication style and started to address John in a more personal manner: “I realize that you think this is taking too long, John” (line 12). However, he still did not explore, or even name, the patient's emotions but continued in a calm and patient way of talking with long pauses (lines 15 and 22), calm responses to profanities (line 21), and a somewhat downplayed way of expressing hope (“our plan is not you will die,” line 25).

The literature on handling emotions in medical consultations, including the NURSE model, tends to emphasize the value of explicit empathic statements and on exploring patients’ emotions (Baile et al., 2000; Pollak et al., 2007; Smith & Hoppe, 1991; Zimmermann et al., 2011). Rather than exploring emotions or giving empathic statements, the oncologist in case A contained the emotions without commenting on the emotion.

The oncologist applied a *containment* strategy, as described by Back and Arnold (2013). The authors point out that patients in emotional chaos may display an emotional volatility, which when started, may spiral out of control. Exploration of emotion may actually trigger resistance. An alternative strategy is an enduring patience and containment of emotions as seen in case A. Similarly, Morse (2001) suggested that doctors should patiently respect but not actively explore the suppressed emotions of patients which are expressed indirectly through profanities and sarcasm, as was the case with the patient in case A.

A containment strategy such as the one the oncologist in case A used is rarely described or discussed in the communication literature in the field of somatic medicine. However, in psychotherapy theory the therapist's tolerance of and patience with the patient's emotions referred to as “containing” is recognized as an important part of the psychotherapeutic relationship (Brown, 2012). Back and Arnold (2013) hold that containment is often required in patient groups that have “high incidence of emotional volatility, low self-regulation, and high impulsiveness” (p. 1431). AYA patients fit well into such a description because they are developmentally prone to behave in a less-controlled manner than adults (Arnett, 2014; Colver & Longwell, 2013).

### Encourage commitment to treatment

In most medical consultations, the doctor and the patient agree about treatment and establish common ground without explicitly saying so (Syse, 2009). Some argue that the fact that a patient is at the hospital talking to the doctor is a form of consent to treatment (Syse, 2009). The final step in SPIKES is described as “strategy and summary” and its aim is to provide the patient with a clear plan for the future to reduce unnecessary uncertainty and anxiety (Baile et al., 2000). In our two cases, the oncologists not only summarized at the end of the consultations, they promoted acceptance of treatment throughout the consultations, eliciting statements of consent from the patients: “I'm in” (line 31) and “I think so, I will get through this” (lines 84–84). In case A, the oncologist used the impersonal “we” when addressing the patient as “you” to encourage commitment to treatment “we need you to be up for it.” In case B, the oncologist did not perform any explicit interactive task to encourage commitment to treatment. The way in which the both oncologists seemed to follow the patients’ lead (Bousquet et al., 2015; Mack & Grier, 2004) and tailored their empathic responses and provision of medical information to the particular AYA patient with whom they were dealing seem equally important to achieving consent to the proposed treatment. By promoting an acceptance throughout the consultation, the oncologists displayed a communicative strategy that in addition to the content level of communication (to get a consent form the patient) also acknowledges that this consent has a relationship aspect (Watzlawick et al., 1967).

### Provide hope

The finale communicative strategy, to provide hope, found in our case study is also associated with the S for strategy (and summary) in SPIKES. The interactive task performed in this strategy was to present optimistic projections. Optimistic projections can be misplaced if they are delivered prematurely or in a way not appropriate for the individual patient (Back, Arnold, Baile, Tulsky, & Fryer-Edwards, 2005; Ryan et al., 2005). Conversely, the oncologists in the two cases studied seemed to tailor their optimistic projections to the patients’ emotional displays described by Morse (2001) as two distinct emotional states (enduring and emotional suffering) (see above, Morse). In case A, the oncologist used WE, but added YOU to make it more personal: “Our plan is *not* that you will die” (line 25) and “it's our goal … you will get well” (lines 28–29). The oncologist did not make any promises he could not keep, but he said that it was not their plan that the patient should die. In case B, on the other hand, the oncologists used a number of expressions to promote hope, for example, “So there is a big chance…” (line 70). She was more personal, using I, than the oncologist in case B in conveying hope according to the emotional suffering state of the patient.

The oncologists tailored their empathic response to the emotions expressed by the AYA. Because of the strong and salient nature of the emotional responses in these two consultations, the oncologists adjusted their communication to the patients’ emotional behavior throughout the consultation, but with more emphasis on acknowledging and containing rather than exploring them. Our results illustrate how expressions of empathy can be tailored to the individual patient and to the nature of his or her emotional response (anger in case A; sadness in case B) when delivering bad news to AYAs.

By tailoring their communicative behavior in the interactive tasks described above, the oncologists define their relationship with the patient. Sometimes the tailoring is done with words or other active behavior. At other times, it is done more passively by showing patience and allowing long pauses. This finding illustrates how all behavior is communication, also a long pause (Watzlawick et al., 1967).

## Limitations and strengths

This study is based on two purposefully sampled consultations, in which we analyzed oncologists’ communication with AYA patients who displayed strong emotions. It would be interesting to see if similar communicative behaviors are used in a larger sample of similar bad news, highly emotive consultations with patients from different age groups. The main limitation of our study is that the analysis is based on our interpretations of our observations; we do not know if the oncologists or the patients would agree with our account. Interviews with the parties involved in the consultations would be a useful supplement to our observations and would increase the validity of our interpretations. It would also be interesting to let the patients and oncologists listen to the audiotapes and give us their interpretation of what was going on.

We based our analysis on audio-recordings of consultations, thus limiting analysis to audible aspects of the communication. Although video-recording the consultations would have provided richer information, we deemed it more considerate and less intrusive to use audio recording in such a critical situation.

We have neither considered the family members’ or nurses’ roles in the consultations nor the possible effects of the sex of the patients and the oncologists. To do so might have added information to our study but we do not think it would change our conclusions about the oncologists’ communicative behavior.

The main strength of this study is that it is based on audio-recordings of genuine consultations between AYAs and oncologists at the time of cancer diagnosis, and there is little research available on this topic. Furthermore, the two cases provide examples of AYAs expressing strong emotions in ways, which will be familiar to clinicians.

## Conclusion and practical implications

AYAs are more likely than younger and older patients to display strong negative emotions, due to their developmental stage (Arnett, 2014). We suggest that a tailored eliciting of the patient perspective, providing of information, responding to patient's expressions of emotion (acknowledgement and containment), encouragement to commitment to treatment, and providing of hope are communication strategies to use in the delivery of bad news, in these cases a cancer diagnosis. Studies of how oncologists can tailor the delivery of bad news to the individual patient in ways that capture more of the complexity of patients communicative styles than the much used SPIKES protocol (Baile et al., 2000) have been requested (Bousquet et al., 2015). Based on a case study of two real-time consultations, the current study provides such knowledge. The study provides exemplars to clinicians of how communication with AYAs displaying strong emotions may unfold and adds to the knowledge base of the delivery of bad news in oncology in general and in AYA oncology in particular.

Further studies are needed to document general patterns of communication in the delivery of bad news to AYA cancer patients, to investigate the participants’ (patients, parents and health-care providers) experiences and opinions, and to investigate potential associations between communicative behaviors and outcomes such as patient satisfaction and adherence to treatment.
